# Draft Genome Sequence of an Anaerobic, Thermophilic Bacterium, *Thermoanaerobacterium aotearoense* SCUT27, Isolated from a Hot Spring in China

**DOI:** 10.1128/genomeA.00041-14

**Published:** 2014-02-13

**Authors:** Hongxia Ai, Junjie Zhang, Mingjun Yang, Pingru Yu, Shuang Li, Mingjun Zhu, Hui Dong, Shengyue Wang, Jufang Wang

**Affiliations:** a School of Bioscience and Bioengineering, South China University of Technology, Guangzhou, China; b Shanghai—MOST Key Laboratory of Health and Disease Genomics, Chinese National Human Genome Center at Shanghai, Shanghai, China

## Abstract

*Thermoanaerobacterium aotearoense* SCUT27, isolated from a hot spring in China, is a strictly anaerobic, thermophilic bacterium capable of degrading xylan and converting both pentose and hexose to ethanol with high yields. Here, we report the draft genome sequence of SCUT27, which reveals insights into the mechanisms of carbon source coutilization and xylan degradation in this thermophilic microorganism.

## GENOME ANNOUNCEMENT

*Thermoanaerobacterium aotearoense* SCUT27, a Gram-positive, thermophilic, strict anaerobe recently isolated from a hot spring in China, has broad substrate specificity, including xylan, dextran (1), and various sugars, including glucose, cellobiose, xylose, mannose, galactose, and arabinose (2). It has been metabolically engineered and developed as a biocatalyst for the production of ethanol, hydrogen, and l-lactic acid (1–3). In order to better understand the molecular mechanisms contributing to sugar utilization and ethanol production, we sequenced the genome of *T. aotearoense* SCUT27.

Whole-genome shotgun sequencing was performed using the Illumina HiSeq 2000 platform to generate 7,453,844 paired-end reads (insert size, ~200 bp), a ~500-fold coverage of the genome. All high-quality reads were *de novo* assembled using the Velvet package (4), and 60 contigs of >500 bp were generated. Protein-coding sequences (CDSs) were predicted using Glimmer (5) and GeneMarkS (6).

The estimated draft genome size of *T. aotearoense* SCUT27 is 2,810,330 bp, with an average G+C content of 34.86%. The genome contains 2,895 predicted CDSs with an average size of 862 bp, accounting for about 88.77% of the draft genome. Approximately 55.3% of the CDSs are assigned to recognizable functional genes, 5.8% have general function predictions only, and the remaining CDSs encode proteins with unknown functions.

Annotation of the genome revealed 10 genes encoding proteins related to xylose utilization, including one xylose isomerase, one xylulose kinase, and 8 ABC-type xylose transporters. Since sugar transport is important for the utilization of carbon sources, the complement of sugar transporters, especially for xylose, was also examined. A total of 147 genes involved primarily in ABC-type transporters and 29 genes in phosphotransferase system (PTS) transporters were identified, which are 13 and 1, respectively, more than those found in *Thermoanaerobacter* sp. strain X514 (GenBank accession no. CP000923.1), a well-studied strain with high xylose utilization capacity (7). Compared to *Thermoanaerobacterium thermosaccharolyticum* DSM 571 (8), SCUT27 possesses two more d-xylose ABC transporter-related genes. Moreover, *T. aotearoense* SCUT27 can degrade xylan directly. Its genome contains one gene for β-1,4-xylanase, one for β-xylosidase, and another 2 for xylanase/chitin deacetylase, which are the key genes responsible for xylan degradation in *Prevotella bryantii* B14 (9).

*T. aotearoense* SCUT27 appears to possess a NADH-dependent butanol dehydrogenase gene. This gene has not been described in other members of the *Thermoanaerobacterium*  genus.

The genomic sequence analysis of *T. aotearoense* SCUT27 provides new insights into the coutilization of glucose and xylose, which can guide future studies of hemicellulose utilization by this organism for biofuel production.

### Nucleotide sequence accession numbers.

This whole-genome shotgun project has been deposited at DDBJ/EMBL/GenBank under the accession no. AYSN00000000. The version described in this paper is version AYSN01000000.
